# Flipping the Card With Enantiodivergent Organocatalysis

**DOI:** 10.1002/chem.71154

**Published:** 2026-05-23

**Authors:** Debora Iapadre, Alessio Carioscia, Alessandro Brusa, Armando Carlone

**Affiliations:** ^1^ Department of Physical and Chemical Sciences Consorzio C.I.N.M.P.I.S., INSTM RU Università degli Studi dell'Aquila L'Aquila Italy

**Keywords:** achiral stimuli, enantiodivergence, organocatalysis

## Abstract

This minireview focuses on recent and most impactful contributions in enantiodivergent organocatalyzed reactions. Four different approaches based on achiral stimuli were examined as inversion strategies: solvent‐controlled inversion, achiral reagent or additive‐inducted inversion, and minimal catalyst modification, discussing in detail for each example the mechanistic insights behind the chiral inversion. Other strategies to achieve enantiodivergence are also briefly discussed.

## Graphical abstract

This minireview elaborates on recent organocatalytic strategies for achieving enantiodivergence—the ability to access both product enantiomers using a single chiral catalyst. It highlights how achiral stimuli, such as solvent polarity and chemical additives, along with minimal catalyst modifications, trigger stereochemical inversions in reactions reported from 2018 to date. By analyzing detailed mechanistic insights, the underlying noncovalent interactions required to shift the field from serendipitous discovery toward the rational design of enantiodivergent methodologies are discussed.

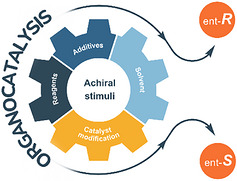

## 1 Introduction

Enantiodivergence is a powerful concept in asymmetric catalysis that enables access to both enantiomers of a chiral product from a single enantiomer of a chiral catalyst [1]. Because nature is inherently homochiral, obtaining the opposite catalyst enantiomer is often challenging or impractical [2]. Enantiodivergent catalysis overcomes this limitation by inverting enantioselectivity through controlled modifications of reaction parameters, such as solvent, temperature, additives, or substrate design, without altering the catalyst's absolute configuration [3]. The significance of enantiodivergent catalysis has been recognized in comprehensive reviews covering both metal‐ and organocatalyzed systems, as well as in discussions of switchable catalysts capable of delivering opposite stereochemical outcomes under controlled conditions [1, 3, 4, 5, 6, 7]. Recently, Y. Hayashi provided an insightful perspective related to enantiodivergent transformations, focusing on stereochemical complexity and condition‐dependent stereocontrol related to central and axial chirality [8]. Despite the growing number of enantiodivergent transformations, no recent (post‐2018) review has focused exclusively on organocatalytic approaches. Asymmetric organocatalysis [9, 10] with its broad diversity of catalyst architectures and activation modes [11, 12, 13, 14], provides a highly versatile platform for stereoselective synthesis, both for academic [15, 16] and industrial applications [17, 18, 19, 20, 21]. This minireview aims to address recent updates on enantiodivergent organocatalysis by providing a focused and critical overview from 2018 to the present. Each section will examine a different inversion strategy, such as solvent change, addition of achiral additives, variation of an achiral reagent, or minimal modifications of catalyst structure, highlighting milestone studies alongside innovative, recent methodologies that enable the enantiodivergent construction of complex structural motifs. In selecting the examples discussed below, approaches requiring the prior modification of starting materials were excluded. An elegant alternative for achieving enantiodivergence involves the photo‐isomerization of alkenes to unlock distinct reaction pathways; for further insights into this specific topic, the reader is directed to the relevant literature [22, 23]. Mechanistic insights into the structural and environmental factors that drive enantioselectivity inversion will be emphasized, with the aim of guiding the rational design of future organocatalysts and methodologies.

## 2 Solvent‐Controlled Inversion of Enantioselectivity

Solvent choice is a key determinant of both reaction outcome and enantioselectivity in asymmetric catalysis. These effects arise from the ability of solvents of different polarity to differentially organize substrates and catalysts within the transition state, thereby modifying the network of noncovalent interactions that govern stereochemical control. In noncovalent organocatalysis, where high enantioinduction depends on the formation of a well‐defined chiral intermediate, such solvent‐dependent organization can profoundly alter the stereochemical course of the reaction. In this context, the role of the solvent in the inversion of enantioselectivity in organocatalysis has been deeply studied. For instance, it is reported that the activity of several organocatalysts is deeply influenced by the solvent polarity, both in Mannich‐type, aldol, (sulfa)Michael, and chlorocyclization reactions [24, 25, 26, 27, 28]. Such solvent effects often reflect a shift in the thermodynamic nature of the stereodetermining transition state. In this context, an interesting review by Rana and coworkers on solvent‐controlled stereodivergent catalysis emphasizes the pivotal role of solvent choice in modulating the enantiomeric outcome of a reaction [29]. In polar protic solvents, enantioselectivity is often enthalpically controlled: strong solvent–substrate or solvent–catalyst hydrogen bonding can selectively stabilize one transition state over the other, lowering its activation enthalpy (Δ*H* ^‡^). In contrast, apolar or weakly interacting solvents often lead to entropic control, where the favored pathway is determined by differences in conformational flexibility or pre‐organization, thus minimizing entropic cost (Δ*S* ^‡^) in one pathway over another. The different (enthalpic or entropic) control of the reaction depending on the solvent can be different enough to reverse the sense of stereoinduction. This effect can be analyzed through a detailed kinetic analysis based on the Eyring Equation (1), which is equivalent to Equation (2), whereas ΔΔ*S* ^‡^_S−R_ and ΔΔ*H* ^‡^_S−R_ denote the differential activation entropy and enthalpy values associated with the transition states leading to the (*S*)‐ and (*R*)‐ enantiomeric products [30, 31, 32].

(1) $$\;\ln\left(\frac{k_{S}}{k_{R}}\right)=-\frac{\Delta \Delta H_{S-R}^{‡}}{RT}+\frac{\Delta \Delta S_{S-R}^{‡}}{R}$$

(2) $$\;\Delta \Delta G_{S-R}^{‡}=\Delta \Delta H_{S-R}^{‡}\;-\;T\Delta \Delta S_{S-R}^{‡}.$$

**SCHEME 1:**
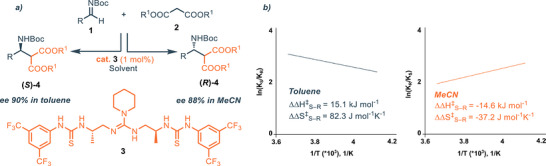
(a) Enantiodivergent Mannich‐type reaction of aldimines (**1**) with malonates (**2**) catalyzed by **3**. (b) Eyring plot for the reaction in toluene (blue line) and in acetonitrile (orange line), adapted from ref [33].

From the value of the Gibbs free energy difference between the transition states leading to the two enantiomers (ΔΔ*G* ^‡^_S–R_), it is possible to determine the preferred stereochemical pathway: when ΔΔ*G* ^‡^_S–R_ > 0, the pro‐(*R*) transition state is favored; conversely, when ΔΔ*G* ^‡^_S–R_ < 0, the reaction preferentially proceeds through the pro‐(*S*) transition state. By plotting the natural logarithm of the ratio between the formation rates of the *R* and *S* enantiomers, ln(*k*_S_/*k*_R_), against the inverse of the absolute temperature (1/T), a linear relationship is obtained according to the Eyring equation. From this linear fit, the enthalpic (ΔΔ*H* ^‡^_S–R_) and entropic (ΔΔ*S* ^‡^_S–R_) contributions to enantioselectivity can be extrapolated: the slope corresponds to ΔΔ*H* ^‡^_S–R_/R, and the y‐intercept to ΔΔ*S* ^‡^_S–R_/*R*. In the case of enantiodivergent reactions, different solvents or conditions typically lead to plots with distinct slopes and intercepts, reflecting a switch in the dominant stereocontrolling factor, from enthalpic to entropic, or vice versa. For example, in 2010, Nagasawa and coworkers reported the enantioselective Mannich‐type reaction between aldimines and diketones catalyzed by bis‐thiourea catalyst **3** [24]. The authors reported that the enantiomeric outcome of the reaction could be reversed depending on the solvent polarity (Scheme 1a). Specifically, in nonpolar solvents such as toluene, the (*S*)‐enantiomer of **4** was preferentially formed, while in polar aprotic solvents like acetonitrile, the (*R*)‐enantiomer became dominant. Eyring plot analysis and extraction of the ΔΔ*H* ^‡^_S–R_, ΔΔ*S* ^‡^_S–R_, and ΔΔ*G* ^‡^_S–R_ parameters revealed that both enthalpic and entropic factors contribute to stereoselection. However, in toluene, the pro‐*S* transition state was predominantly influenced by entropic effects, whereas in MeCN, the enantioselectivity favouring the pro‐(*R*) pathway was primarily driven by enthalpic control (Scheme 1b).

**SCHEME 2:**
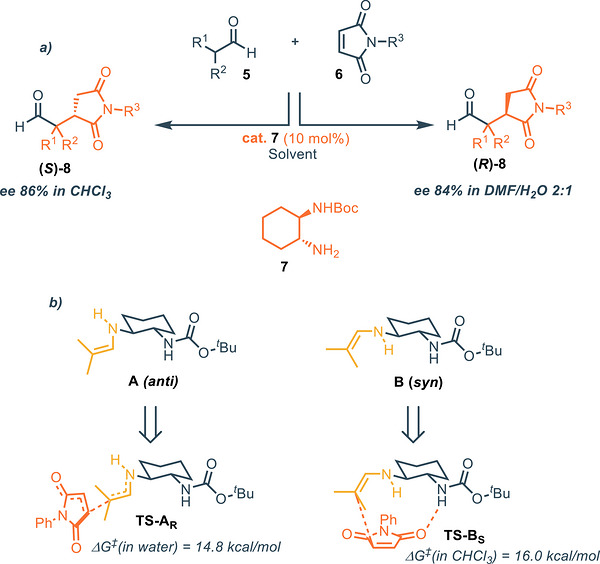
(a) Enantiodivergent addition of aldehydes **5** to succinimides **6** catalyzed by **7**. (b) Enamine conformation **7** and relative computed TS structures.

The effect of the solvent can also be rationalized using Density Functional Theory (DFT) computational calculations by evaluating the relative stabilities of competing transition state (TS) structures in solvents of different polarity. Typically, nonpolar solvents tend to favour transition states stabilized by intramolecular noncovalent interactions (e.g., hydrogen bonding), which are less disrupted in such media. In contrast, polar solvents preferentially stabilize polar transition states via solvation, thereby altering the spatial arrangement of the reactants in the TS and possibly leading to opposite enantioselectivities. In this regard, Chinchilla, Gómez‐Bengoa, and coworkers reported that catalyst **7** could catalyse the enantioselective addition of α‐branched aldehydes to maleimides. Notably, the stereochemical outcome of this reaction was strongly dictated by the solvent. While CHCl_3_ provides the desired **(*S*)‐8** in 86% ee, a mixture of DMF/H_2_O 2:1 delivered the opposite (*R*)**‐8** enantiomer in −84% ee (Scheme 2a). The authors explained this solvent‐dependent reversal of enantioselectivity mainly through DFT calculations. They modelled the intermediate enamine and identified two energetically favourable conformations (A and B). For conformation A, where the two NHs are in an *anti*‐relative orientation, the transition state TS‐A_R_, where the enamine attacks the Si‐face of the maleimide, was found to be highly polar and had the lowest activation barrier in water (Δ*G* ^‡^ ≈ 14.8 kcal/mol), leading to the (*R*)‐enantiomer. In contrast, for conformation B, where the two NHs are oriented in a *syn*‐fashion, the corresponding transition state TS‐B_S_ was less polar and more stabilized in chloroform (Δ*G* ^‡^ ≈ 16.0 kcal/mol), due to reduced charge separation and favourable internal noncovalent interactions, thus favouring the (*S*)‐enantiomer (Scheme 2b) [34]. Later on, the same group demonstrated that a similar catalyst could perform the same reaction, retaining the enantiodivergent mechanism, albeit with a higher enantiomeric induction [35].

It becomes evident that the two analysis methods (experimental kinetic analysis based on the Eyring equation vs. DFT calculations) provide different, although complementary, information. While Eyring's analysis delivers global thermodynamic insights without showing any structure or geometries of the TS, DFT calculations predict the Δ*G* ^‡^ and practically show how the reactants are arranged in the TS but rely on simplified models that are sensitive to the level of theory and to the solvation model.

**SCHEME 3:**
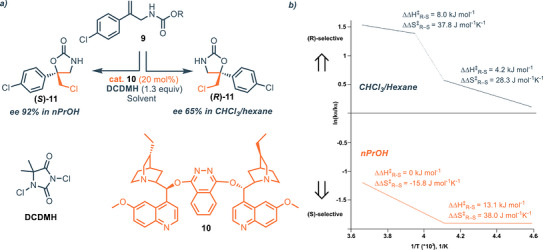
(a) Enantiodivergent chlorocyclization of unsaturated carbamate **9** catalyzed by **10**. (b) Eyring plot for the reaction in CHCl_3_/hexane (blue line) and in n‐propanol (orange line), adapted from ref [27].

A clear example of this is found in the enantiodivergent chlorocyclization of unsaturated carbamates **9**, catalyzed by (DHQD)_2_PHAL catalyst **10**, as described by Borhan and coworkers (Scheme 3a) [27]. In this system, a solvent‐induced inversion of enantioselectivity is observed: the reaction proceeds with high selectivity toward the **(*R*)‐11** enantiomer in nonpolar solvents such as CHCl_3_/hexanes, whereas polar protic solvents like n‐propanol favour the (*S*)‐enantiomer **(*S*)‐11**. Crucially, the authors report the presence of inflection points in the Eyring plots, indicating that different transition states become dominant at different temperature regimes. This behavior implies a mechanistic switch, likely involving competing pathways with different sensitivities to enthalpic and entropic contributions. Under these conditions, kinetic analysis becomes a particularly powerful tool, as it can detect these mechanistic shifts by analysing how the enantiomeric ratio varies with temperature. For instance, in CHCl_3_/hexanes, the selectivity increases with temperature, suggesting that entropic factors dominate and favour a more disordered, possibly less structured transition state. Although both ΔΔ*H* ^‡^ and ΔΔ*S* ^‡^ remain positive across the temperature range, the entropic contribution to ΔΔ*G* ^‡^ (T·ΔΔ*S* ^‡^) becomes smaller at lower temperatures, reducing the overall free energy difference between the competing transition states. As a result, the enantioselectivity in favor of the (*R*)‐product diminishes. In contrast, in n‐propanol, the selectivity decreases with increasing temperature, pointing to a transition state stabilized by specific enthalpic interactions (e.g., hydrogen bonding) at lower temperatures (Scheme 3b). These divergent trends are difficult to capture accurately with DFT alone, due to the complexity of modelling explicit solute–solvent and conformational effects across a wide temperature range.

**SCHEME 4:**
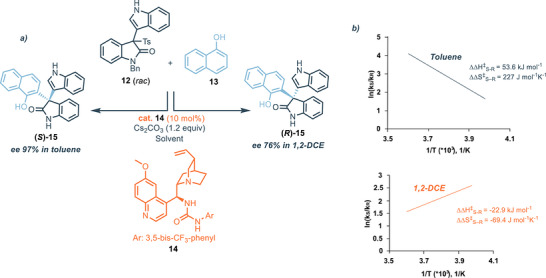
(a) Enantiodivergent Friedel–Craft reaction of sulfonyl indole **12** with 1‐naphthol **13** catalyzed by **14**. (b) Eyring plot for the reaction in toluene (blue line) and in 1,2‐dichloroethane (orange line), adapted from ref [36].

DFT calculations and Eyring kinetic analysis were jointly employed to elucidate the solvent effect in the enantiodivergent Friedel–Crafts reaction between sulfonyl indoles **12** and 1‐naphthols **13**, catalyzed by the quinine–urea bifunctional catalyst **14** (Scheme 4a) [36]. As demonstrated, the solvent polarity plays a crucial role in controlling the enantioselectivity. While nonpolar solvents (e.g., toluene, *m*‐xylene, CHCl_3_) afford the **(*S*)‐15** adduct with good to excellent enantioselectivity (up to 97% ee in toluene), polar aprotic solvents such as 1,2‐dichloroethane (1,2‐DCE) give the (*R*)‐enantiomer (−76% ee). The Eyring plots of ln(*k_S_/k_R_*) versus 1/*T* were modelled to extract the differential activation parameters (ΔΔ*H* ^‡^ and ΔΔ*S* ^‡^) based on the Eyring equation (Equation 1). These plots revealed that in nonpolar solvents, the enantioselection is entropy‐driven, whereas in polar solvents, the selectivity is predominantly enthalpy‐controlled (Scheme 4b). Structurally, DFT calculations suggest that the transition states differ in their hydrogen‐bonding geometry: the (*S*)‐selective TS in toluene shows longer, weaker H‐bonds, consistent with a more flexible, entropy‐driven transition state, while the (*R*)‐selective TS in 1,2‐DCE features shorter, stronger H‐bonds, indicative of a more rigid, enthalpy‐controlled pathway. These energetic and geometric differences align well with the experimental enantioselectivities observed under each solvent condition.

**SCHEME 5:**
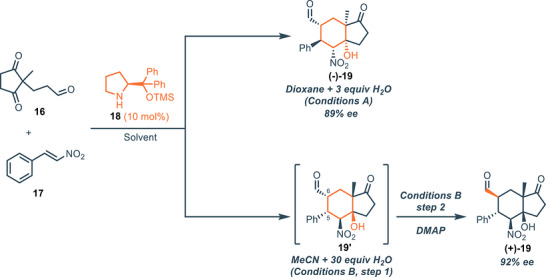
Enantiodivergent Michael–Henry domino reaction for the construction of Hajos–Parrish‐type ketones.

Recently, Hayashi and coworkers reported the enantiodivergent synthesis of Hajos–Parrish ketone (HPK) analogues via a domino Michael–Henry reaction catalyzed by diarylprolinol organocatalyst **18** (Scheme 5). A key finding was that the amount of water in the solvent mixture dictates a complete inversion of all five contiguous stereocentres. When the reaction was carried out in dioxane with 3 equivalents of water (conditions A), compound (−)−**19** was obtained with excellent enantioselectivity. In contrast, performing the reaction in MeCN with 30 equivalents of water (conditions B, step 1) furnished the diastereomer **19**′ as the kinetic product, again with excellent enantiocontrol. Subsequent treatment with 10 mol% DMAP (conditions B, step 2) induced epimerization at C6 of **19**′, affording (+)−**19** in high yield and enantioselectivity. The origin of the overall enantioselectivity reversal lies in the diastereoselectivity during the Michael addition of the enamine on nitrostyrene, which establishes the configuration at C5 and thereby controls the subsequent stereochemical cascade. Under conditions A, the enamine reacts with the *Re*‐face of nitrostyrene to give (−)−**19**, whereas under conditions B (step 1), the *Si*‐face attack predominates, leading to **19**′. Thus, (−)−**19** is the kinetic product under conditions A, while **19**′ is the kinetic product under conditions B. Epimerization of **19**′ at C6 under basic conditions (step 2) converts it into the thermodynamically more stable (+)−**19** [8, 37]. The authors could not support the mechanistic hypothesis via computational methods, as the *inherent complexity of the system* prevented the identification of any transition state.

## 3 Achiral Reagent or Additive‐Inducted Inversion

Enantiodivergence can also be induced by modification of an achiral reagent by using different achiral sources. Reagents and additives, such as acids or bases, can affect the transition state, especially during the formation of noncovalent interactions like hydrogen bonds, or in the case of steric and electronic contributions.

As extensively discussed in a comprehensive review by Beletskaya et al. [3], in 2016, Akiyama developed the enantiodivergent synthesis of axially chiral molecules by dynamic kinetic resolution (DKR) in the presence of chiral phosphoric acids (CPAs). Axially chiral molecules are both important pharmaceutical compounds [38] and widespread components since they are used as chiral ligands for metal or organocatalysts. Akiyama showed that the reductive amination under DKR conditions by means of CPAs proceeded with excellent enantioselectivities to furnish both enantiomers of chiral biaryls, based on the position of substituents on hydroxyaniline derivative [39].

**SCHEME 6:**
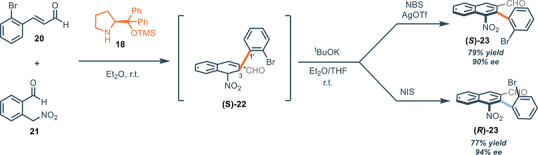
One‐pot organocatalyzed enantiodivergent synthesis of axially chiral biaryl compound **23**.

In recent years, similar enantiodivergent strategies for the synthesis of axially chiral molecules have gained more interest. Methods based on different achiral reagents or additives provide access to (*R*) and (*S*) isomers. In 2021, Hayashi and coworkers reported the enantiodivergent one‐pot synthesis of chiral biaryls using a diarylprolinol (Scheme 6) [40]. A Michael/aldol domino reaction allows the conversion from central to axial chirality, providing an alternative and valuable strategy for the construction of axial chiral compounds. Enantiodivergence arises from a defined interplay between halogenated sources and additives.

Prolinol silyl ether **18** catalyzed the Michael/aldol domino reaction between **20** and **21**, respectively, via iminium ion (Michael addition) and via enamine (aldol reaction). Compound **23** was obtained as (*R*) and (*S*) isomers in good yield and with excellent diastereo‐ and enantioselectivities. To convert the central chiral compound **(*S*)‐22** to axial chiral product **23**, the mechanism consists of 3 steps: (1) nitronate formation in the presence of t‐BuOK; (2) halogenation of the nitronate using N‐halosuccinimide (NXS); 3) aromatization process by elimination of HX. All the steps involved can be carried out in a one‐pot fashion. The authors discovered that enantiodivergence is strongly related to the stability of the halogenated intermediate and the acid–base character of the additives used. To deeply understand the mechanism responsible for the enantiodivergence process, intermediates **24a** and **24b** were prepared using NBS and NIS as halogen sources, respectively. Both **24a** and **24b** were isolated as single diastereoisomers, sharing the same configuration of the C3─C1’ bond. Nevertheless, **24a** is more stable and less reactive than **24b** at room temperature. Consequently, the conformation was determined for **24a** by x‐ray crystallographic analysis and assumed by analogy for **24b**, given their closely similar spectrum. Both intermediates are treated with K‐succinimide. In this case, the reaction proceeds smoothly with **24b** providing **(*R*)‐23** with high ee, while no reaction occurs with **24a** (Table 1, entries 1 and 2). Intermediate **24a** furnishes **(*R*)‐23** only in the presence of stronger bases (entries 3 and 4). On the other hand, both **24a** and **24b** give the desired product with (*S*) configuration in the presence of Ag salts like AgOTf (entries 5 and 6).

**TABLE 1:** The reactivity of **24a** and **24b** in the presence of different additives.

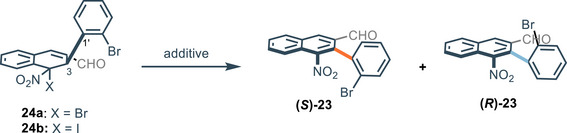			
Entry	Intermediate	Additive	ee%
1	**24b**	K‐succinimide	96 (**(R)‐23**)
2	**24a**	K‐succinimide	n.d.
3	**24a**	^t^BuOK	>98 (**(R)‐23**)
4	**24a**	Et_3_N	>98 (**(R)‐23**)
5	**24a**	AgOTf	94 (**(S)‐23**)
6	**24b**	AgOTf	92 (**(S)‐23**)

**SCHEME 7:**
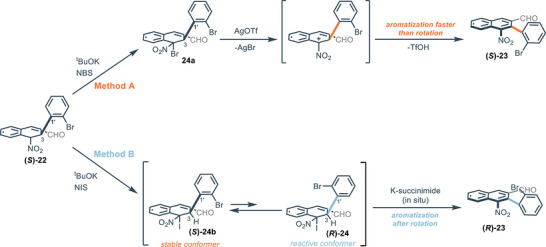
Proposed mechanism for enantiodivergent synthesis of **23**.

The proposed mechanism for the two pathways is supported by the agreement between DFT calculations and experimental evidence (Scheme 7).

In Method A, treatment of **24b** with AgOTf leads to the formation of a cationic intermediate that is highly unstable due to the presence of an adjacent NO_2_ group. In this scenario, deprotonation would proceed rapidly before rotation around the C3─C1’ bond, resulting in the formation of **(*S*)‐23**. In Method B, there is no formation of a cationic intermediate, and the mechanism proceeds as synperiplanar E2 elimination, mediated by K‐succinimide produced in situ. Between the two possible conformers shown in Scheme 7, **(*S*)‐24b** was found to be the most stable at room temperature, both from DFT calculation and experimental analysis (**(*S*)‐24b** is the only one observed by ^1^H‐NMR). The enhanced stability of **(*S*)‐24b** toward elimination arises from the steric hindrance, which disfavors the deprotonation at C3. On the other hand, the conformation of ***(R)*‐24** would make the proton on C3 more available. The rotational barrier of the C3─C1’ bond was calculated to be 74.11 kJ/mol, indicating that **(*S*)‐24b** can easily interconvert to **(*R*)‐24** at room temperature. Thus, selective deprotonation at C3 would proceed from ***(R)*‐24**, leading to **(*R*)‐23** with excellent enantiopurity.

**SCHEME 8:**
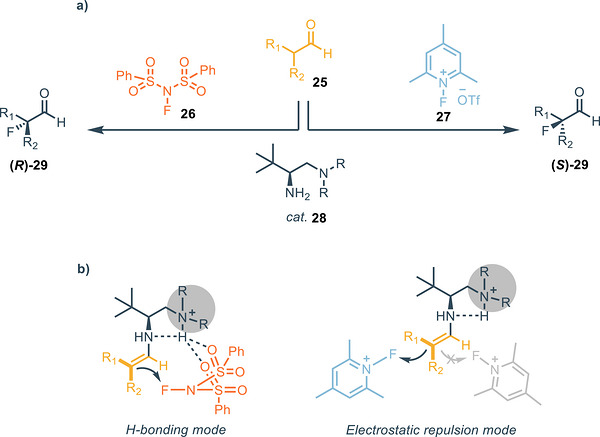
(a) Organocatalyzed fluorination of α‐branched aldehydes, an example of reagent‐controlled enantiodivergent synthesis; (b) proposed transition states related to hydrogen‐bonding mode (*Re*‐facial attack) and electrostatic repulsion mode (*Si*‐facial attack).

Hydrogen bonding and electrostatic interactions between the organocatalyst and achiral reaction partners are key factors in the induction of enantiodivergence. Enamine catalysis was shown to provide effective access to both enantiomers of α‐fluoroaldehydes **29**, by selecting the fluorinating agent used [41]. In combination with an acid co‐catalyst, chiral primary‐tertiary diamine **28** was used as an organocatalyst for asymmetric fluorination of α‑branched aldehydes (Scheme 8a). Primary amines act as catalysts with α‐substituted aldehydes **25** via enamine catalysis. The chiral primary amine reacts with the aldehyde to form the *E*‐enamine intermediate prevalently. Compound **26** or **27** leads to the formation of opposite enantiomers based on, respectively, hydrogen‐bonding mode or electrostatic‐repulsion mode; indeed, the solvent also plays an important role in the reaction outcome.

The authors found that the (*R*)‐selective process is favored with neutral fluorination reagent **26** in aprotic and low‐polar solvents. In CHCl_3_, **(*R*)‐29** is obtained in 86% of yield and 85% ee, while the enantioselectivity drastically drops in MeOH. This supports the H‐bonding mode (Scheme 8b), because solvent effects and competing interactions are minimized in a low‐polar solvent like CHCl_3_. On the other hand, cationic fluoro reagent **27** favored (*S*)‐selective process in polar and protic solvent; in DMF, **(*S*)‐29** is obtained in 82% of yield and 85% ee, while **27** leads to **(*R*)‐29** with modest ee in CHCl_3_. The author concluded that this behavior could be explained by an electrostatic repulsion mode (Scheme 8b); notably minor modifications on catalyst **28** lead to a concomitant increase in selectivity. Bulkier substituents, like isopropyl, on the tertiary amine group favor the electrostatic repulsion mode by steric hindrance.

**SCHEME 9:**
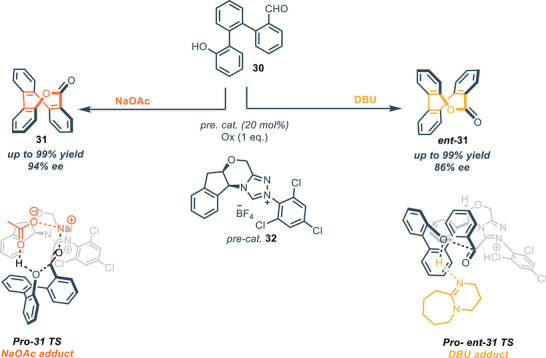
Enantiodivergent carbene‐catalyzed intramolecular cyclization of triaryl aldehydes to access inherently chiral lactones. The enantioselective switch is under base control.

More recently, enantiodivergent synthesis of eight‐membered lactones with inherent chirality was achieved through carbene‐catalyzed base‐controlled synthesis, in which enantiodivergence was governed by the formation of distinct noncovalent interactions depending on the reagent employed [42]. Specifically, different lactones were prepared, and the authors demonstrate that one enantiomeric form exhibits remarkable antimicrobial and antibacterial properties in an agricultural field. The transformation is an intramolecular cyclization of triaryl aldehyde **30** under basic conditions (Scheme 9). The reaction proceeds with excellent yield and selectivity, and surprisingly, achiral bases induce enantiodivergence. The best results are obtained in the presence of NaOAc and DBU. The robustness of the procedure is supported by a wide scope considering electron‐donating and electron‐withdrawing groups and different substituent positions on triaryl aldehyde aromatic rings. In most cases, the product can be obtained in both enantiomeric forms, in high yield and enantioselectivity.

The authors performed DFT calculations to understand the base‐controlled enantiodivergent mechanism [42]. Calculations highlight that different bases have completely different interactions during the cyclization step of compound **30**. The first step corresponds to the formation of carbene‐catalyst in‐situ from its precursor **32**. Carbene‐catalyst reacts with **30** to form an acyl azolium intermediate, which can exist in different conformations. The lowest energy is associated with conformers where the hydroxyl group is close to the carbonyl carbon of the acyl azolium. Rotation barrier around C─C axial bond is facile, allowing easy interconversion between different conformations. The transition states (TSs) are closely related to how the base performs the deprotonation of the hydroxyl group on substrate **30**. Indeed, phenoxide oxygen would attack the carbonyl group of the acyl azolium intermediate, determining opposite ring closures. Noncovalent interactions and distortion energy seem to be the basis of enantiodivergence. In both cases with NaOAc and DBU, two possible TS structures are considered and, as expected, the one with the lower energy barrier leads to the major enantiomer, in good agreement with the experimentally observed ee. (Scheme 9). In both cases, the lower barrier energy is associated with the high degree of noncovalent interactions, which determine the opposite configuration during ring closure.

**SCHEME 10:**
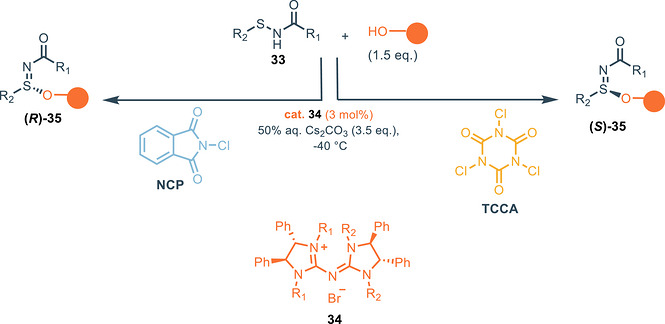
General procedure for the reagent‐controlled enantiodivergent synthesis of sulfinimidate **35** catalyzed by PN catalyst **34**.

Recently, enantiodivergent synthesis of chiral sulfinimidate esters was reported, exploiting a reagent‐regulated organocatalytic procedure [43]. Zhang and coworkers achieve a tandem chlorination and nucleophilic substitution of sulfenamide in the presence of alcohols as nucleophiles (Scheme 10). The reaction is catalyzed by the same enantiomer of chiral pentanidium **34** (PN), which reacts with sulfenamide **33** under basic conditions, resulting in an ion pair.

**SCHEME 11:**
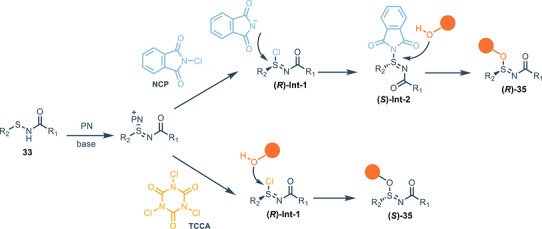
Proposed mechanism for the reagent‐controlled enantiodivergent synthesis of sulfinimidates.

N‐chlorophthalimide (NCP) and trichloroisocyanuric acid (TCCA) are used as chlorinating reagents, leading respectively to (*R*) and (*S*) isomers of the desired product. When employing TCCA, the reaction takes place in only 5 min, whereas 24 h are needed when NCP is used. The authors proposed an enantiodivergent pathway by which the tandem chlorination and nucleophilic substitution take place under reagent control, to explain the different behaviors. In the presence of NCP, **(*R*)‐Int‐1** undergoes two consecutive nucleophilic attacks: the first by phthalimide anion generated in situ, leading to **(*S*)‐Int‐2**; the second by the alcohol on **(*S*)‐Int‐2,** which is slowly converted to **(*R*)‐35** isomer. On the other hand, intermediate **(*R*)‐Int‐1** undergoes direct nucleophilic attack by the alcohol, resulting in inversion of configuration and formation of **(*S*)‐35** when TCCA is used as chlorination source (Scheme 11).

The authors demonstrated the different mechanisms proposed based on experimental evidence. The observed slow reaction rate is consistent with the formation of the stable intermediate **(S)‐Int‐2** when NCP is used as a chlorinating agent. At the beginning of the reaction, a new ^19^F‐NMR signal is detected when the fluorinated analogue of **33** is used; this signal progressively disappears as the concentration of the desired product increases. Furthermore, **Int‐2** was detected using high‐resolution mass spectrometry (HRMS) when NCP derivatives were employed, whereas it was not detected when TCCA was used as a chlorinating source. This observation confirms that the reaction proceeds through a different mechanism and occurs on a significantly shorter timescale.

**SCHEME 12:**
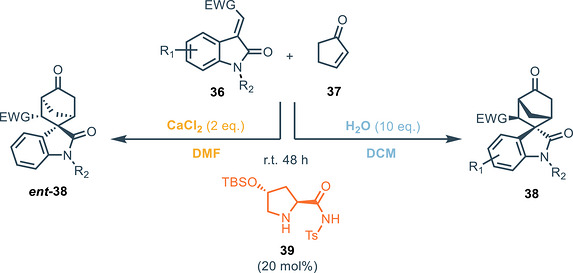
Diastereo‐ and enantioselective organocatalytic [4+2]‐annulation for the synthesis of spirooxindole‐norcamphor scaffold. Enantiodivergence is induced by the presence of CaCl_2_ and DMF or H_2_O as additives.

In 2021, Chen, Yang et al. highlighted the role of additives in the stereoselective organocatalytic [4+2]‐annulation between isatyledenes **36** and 2‐cyclopentenone **37** [44]. The authors reported a synthetic strategy to access both enantiomers of spirooxindole‐norcamphor scaffold **38** by adding either CaCl_2_ and DMF or H_2_O in the reaction media (Scheme 12).

Prolinosulfonamide **39** can catalyse the [4+2]‐annulation reaction in high yield and high diastereo‐ and enantioselectivity for both enantiomers. In one case, the best results are achieved by adding 10 eq. of H_2_O in DCM (Table 2, entry 1). On the other hand, the addition of CaCl_2_ in the same reaction conditions provides a drastic drop of ee (Table 2, entry 2), suggesting that CaCl_2_ would favor the formation of the opposite enantiomer ***ent*‐38**. Indeed, with CaCl_2_ in DMF as a solvent, a complete reverse of enantioselectivity was achieved (Table 1, entry 3), and the reaction is further promoted by the addition of 20 mol% K_2_CO_3_ (Table 2, entry 4). To better understand the role of Ca^2+^, water was deliberately introduced into the reaction mixture. Notably, 0.1 mL of H_2_O was sufficient to suppress the effect induced by CaCl_2_ on enantioselectivity (Table 2, entry 5). In contrast, the addition of 200 µL of DMF in DCM as solvent media afforded ***ent*‐38** with 60% ee (Table 2, entry 6). These results highlight the role of DMF‐calcium coordination for the enantiodivergence induction. The proposed mechanism envisaged prolinosulfonamide **39** as a bifunctional catalyst, activating 2‐cyclopentenone **37** to generate the corresponding chiral dienamine while engaging compound **36** through sulfonamide N─H hydrogen‐bonding interactions.

**TABLE 2:** Optimization of reaction conditions.

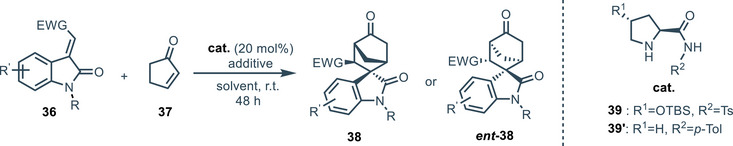					
Entry	Catalyst	Solvent	Additive	Yield %	ee%
1	**39**	DCM	H_2_O (10 eq.)	91	93 (**38**)
2	**39**	DCM	CaCl_2_ (2 eq.)	15	0
3	**39**	DMF	CaCl_2_ (2 eq.)	92	59 (***ent*‐38**)
4	**39**	DMF	CaCl_2_, K_2_CO_3_	94	98 (***ent*‐38**)
5	**39**	DMF	CaCl_2_, 0.1 mL H_2_O	76	50 (**38**)
6	**39**	DCM	CaCl_2_, 200 µL DMF	86	60 (***ent*‐38**)
7	**39’**	DMF	CaCl_2_	69	46 (**38**)

**SCHEME 13:**
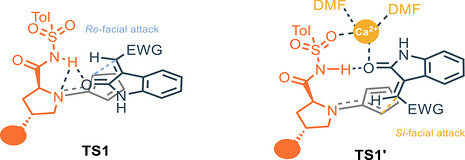
Proposed transition states TS1 and TS1’ in the presence or absence of CaCl_2_ and DMF.

Without CaCl_2_, hydrogen‐bonding interactions involving the sulfonamide group guide the *Re*‐facial attack of dienamine intermediate to the C‐C double bond of compound **36**, leading to **38**. In contrast, CaCl_2_ in the presence of DMF induces a distinct transition‐state arrangement, driven by Ca^2+^ coordinating the sulfonyl group of the catalyst and the oxindole carbonyl group of compounds **36**. In agreement with this interpretation, the reaction catalyzed by catalyst **39**′ in the presence of CaCl_2_ afforded isomer **38** as the desired product (Table 2, entry 7), highlighting the key role of the sulfonyl group in TS1’ stabilization and enantioinduction. (Scheme 13).

**SCHEME 14:**
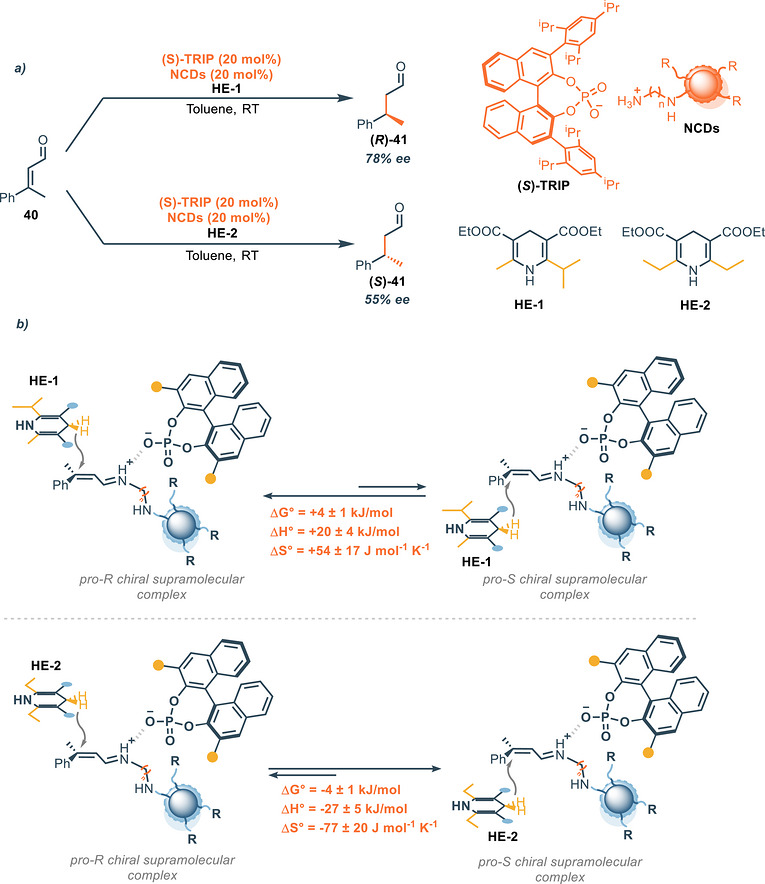
Enantiodivergent nano‐organocatalytic transfer hydrogenation of *α*,*β*‐unsatured aldehydes under ACDC catalysis, upon minimal modification on the HE. (a) Reaction scheme; (b) Relative stability of pre‐reactive complex determined via MD simulations.

Recently, Carlone, Aschi, and coworkers reported a remarkable case of reagent‐controlled enantiodivergence in the asymmetric transfer hydrogenation of unsaturated aldehydes **40**, catalyzed by Nitrogen‐doped Carbon Dots (**NCDs**) under Asymmetric Counteranion‐Directed Catalysis (**ACDC**) conditions [45]. The catalytic system comprises NCDs acting as the iminium‐forming catalyst and a Chiral Phosphoric Acid **(*S*)‐TRIP** with fixed handedness. The authors discovered that the stereochemical outcome is strictly dependent on the substitution pattern of the Hantzsch ester (**HE**) used as the hydride source. While standard HEs (**HE‐1**) provided the expected enantiomer with high selectivity, the use of a symmetrical diethyl‐substituted Hantzsch ester (**HE‐2**) triggered a complete reversal of enantioselectivity, favoring the opposite enantiomer (Scheme 14a). Crucially, control experiments revealed that this inversion is unique to the nanostructured catalyst; when simple molecular amines (e.g., piperidine or morpholine) were used, no such reversal was observed, suggesting that the phenomenon arises from a specific steric interplay within the supramolecular assembly involving the **NCDs** surface. To rationalize this counterintuitive switch, which could not be adequately described by static DFT calculations due to the vast conformational landscape, the authors employed extensive semi‐classical molecular dynamics (MD) simulations. This computational analysis highlighted that the stereodetermining event is not merely the hydride transfer itself, but the thermodynamic stability of the pre‐reactive supramolecular complexes formed by the iminium ion, the **CPA**, and the **HE**. The study demonstrated that weak London dispersion forces differ significantly depending on the **HE** substituents, effectively steering the pre‐organization of these complexes. In the case of **HE‐1**, the pro‐(*R*) assembly is enthalpically stabilized. Conversely, with the diethyl‐substituted **HE‐2**, the modulation of noncovalent interactions shifts the thermodynamic preference toward the pro‐(*S*) assembly (Scheme 14b). This work provides a compelling rationalization for enantiodivergence driven by dynamic pre‐organization and dispersion forces, rather than the common transition state energy difference.

## 4 Minimal Catalysts Modification

Another strategy to achieve enantiodivergent organocatalysis is to introduce peripheral modifications to the catalyst structure without changing its inherent stereochemistry. While the stereochemical outcome of a reaction is usually governed by the diastereomeric transition state complex and therefore by the chirality of the catalyst, secondary noncovalent interactions often play a decisive role. Subtle alterations of these interactions can shift the balance of stereocontrol, ultimately leading to a reversal of enantioselectivity.

**SCHEME 15:**
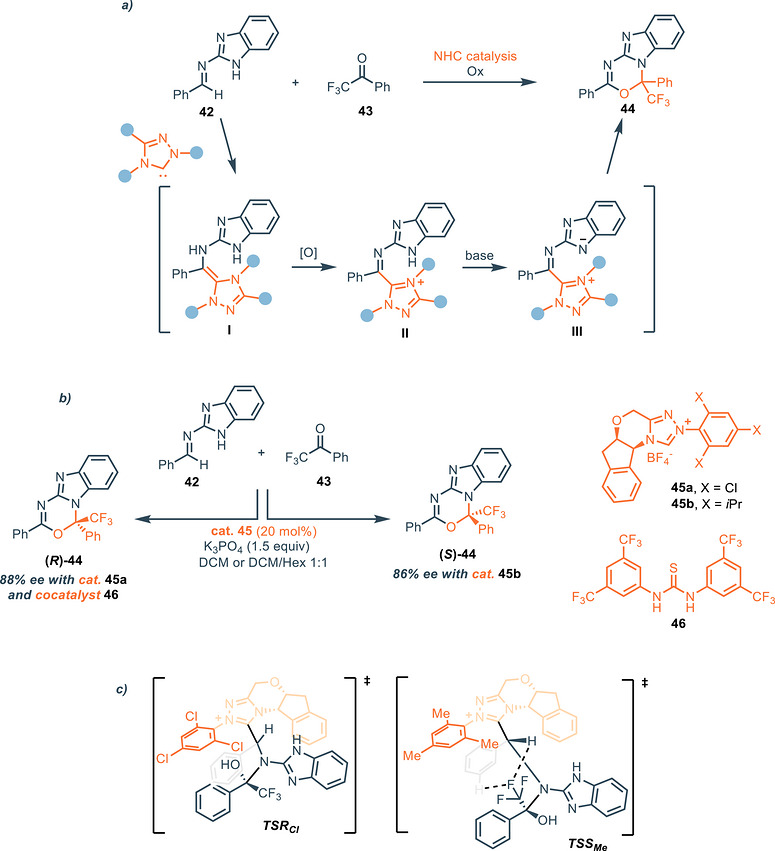
(a) Enantioselective NHC catalyzed [4+2] cycloaddition of aldimines with trifluoromethyl ketones; (b) Minimal modifications of the NHC precursor enable enantiodivergence; (c) Transition states representation (*pro‐S* TS was computed with methyl substituents in the catalyst ring instead of isopropyl substituents).

A prominent example in this regard comes from Huang and coworkers, who reported in 2021 the enantiodivergent NHC‐catalyzed oxidative [4+2] cycloaddition of aldimines with activated ketones (Scheme 15a) [46]. The reaction proceeds via formation of an aza‐Breslow intermediate (**I**) between the NHC and the aldimine **42**, which, upon oxidation, yields intermediate **II**. Subsequent deprotonation generates a 1,4‐dipolar species (**III**), which engages in a [4+2] cycloaddition with trifluoromethyl ketones, furnishing chiral heterocycles with a quaternary carbon center **44**. During the reaction optimization, the author noticed that a subtle modification in the structure of the NHC dramatically affected the sense of enantioinduction. Catalyst **45b**, bearing bulky isopropyl groups on the aryl substituent, afforded the (*S*)‐enantiomer in 86% enantiomeric excess. In contrast, catalyst **45a**, which differs from **45b** only by having chlorine atoms instead of isopropyl groups on the aryl ring, completely reversed the enantioselectivity to 88% ee, favoring the *R*‐enantiomer, when used in conjunction with Schreiner's thiourea **46** as a non‐chiral co‐catalyst (Scheme 15b). This peculiar effect was studied via DFT analysis, revealing that the observed enantioselectivity is governed by noncovalent C─H···F hydrogen bond interactions between the NHC‐substrate complex and the trifluoromethyl ketone. For catalyst **45b**, two stabilizing C─H···F interactions in the transition state favored the formation of the (*S*)‐enantiomer over the *(R)*‐enantiomer, having only one C─H···F interaction. However, the introduction of chlorine atoms in catalyst **45a** disrupted these interactions, leading to an inversion of the favored transition state geometry and thus enantioselectivity. The presence of the thiourea co‐catalyst further stabilized the altered transition state through additional hydrogen bonding, amplifying the selectivity reversal. Notably, since the enantioselectivity reversal is driven by the weakening of C─H···F hydrogen bonds in the stereodetermining transition state, this divergence effect is strictly dependent on the presence of a CF_3_ group in the electrophilic partner. When the substrate lacks a trifluoromethyl substituent, such as in reactions involving isatin derivatives, the noncovalent interactions that control enantioselectivity are no longer operative. As a result, no inversion of enantioinduction is observed, and the reaction proceeds with the inherent selectivity of the parent NHC catalyst.

**SCHEME 16:**
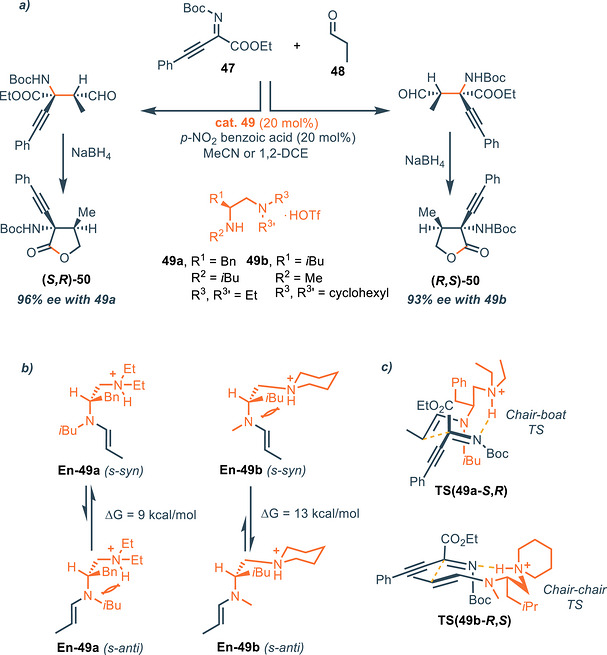
(a) Enantiodivergent Michael addition on C‐alkynyl ketimines; (b) preferred conformation of the different enamines; (c) Most stable TS structures delivering opposite enantiomers with the catalysts **49a** and **49b**.

Another relevant example of this concept is the enantiodivergent Michael reaction between C‐alkynyl ketimines **47** and aldehydes **48** proposed by Shao and coworkers [47]. In this case, chiral secondary amines catalysts were precisely engineered to achieve an enantiodivergent access to chiral propargylamines bearing quaternary stereocentres. In particular, the substitution pattern of the catalyst strongly influences the enantiomeric outcome. When a catalyst featuring a bulky *i*‐Bu group on the secondary amine was used (**49a**), the product **(*S*,*R*)‐50** was formed with excellent diastereo‐ and enantiocontrol. On the other hand, catalyst **49b**, bearing a smaller *N*‐Me group, could catalyse the same transformation, delivering the opposite enantiomer **(*R*,*S*)‐50**, also with high diastereo‐ and enantioselectivity (Scheme 16a). The generality of these two enantiodivergent catalysts was demonstrated by applying the same catalytic systems to a broad range of Mannich‐type reactions involving structurally diverse substrates. DFT calculations were employed to clarify the origin of the enantiodivergence. The resulting data revealed that the enamines derived from **49a** and **49b** feature distinct conformational preferences. For **En‐49a**, the s‐syn is energetically favored (by ∼9 kcal/mol), while for **En‐49b**, the s‐anti conformation is preferred (13 kcal/mol). This is mainly due to different profiles of steric repulsion exerted by the *N*‐substituents within the catalyst structure (Scheme 16b). This way, with catalyst **49a** the *Si* face of the enamine reacts with the *Re* face of the imine, delivering a 9‐membered cycle TS (**49a**‐*S*,*R*) with a chair‐boat arrangement, while catalyst **49b** allows the reaction of the Re face of the enamine with the Si face of the imine, with a chair‐chair arrangement of the transition state TS (**49b**‐*R*,*S*) (Scheme 16c).

**SCHEME 17:**
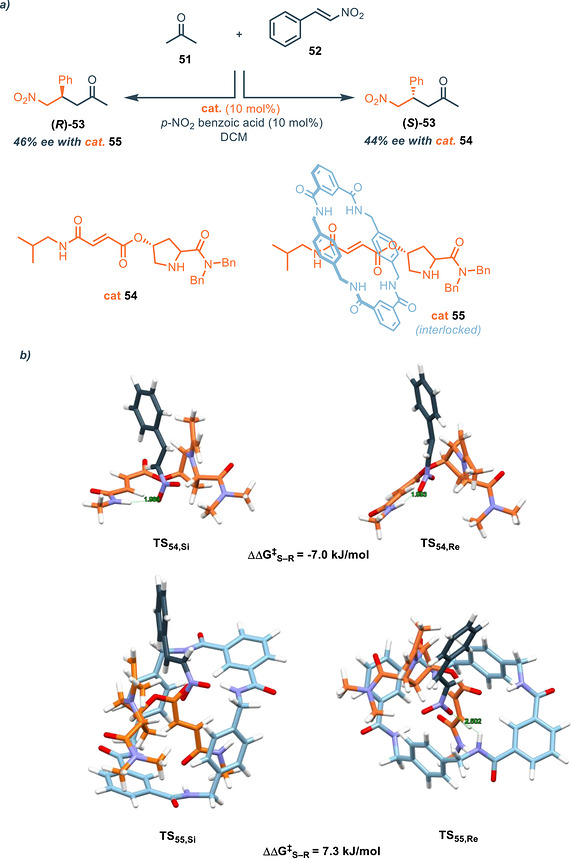
(a) Interlocked and non‐interlocked catalysts **54** and **55** allow for the enantiodivergent Michael addition on nitro olefines; (b) Most stable TS for the attack on the Si‐ and Re‐face of nitrostyrene with catalysts **54** and **55**.

Interlocking the catalyst has emerged as an effective strategy to control, and even reverse, the sense of enantioselectivity in asymmetric catalysis. In 2019, Berna and coworkers reported that the mechanical interlocking of a prolinamide–fumaramide organocatalyst **54** significantly altered the stereochemical outcome of the Michael addition of ketones **51** to *β*‐nitrostyrene **52** (Scheme 17a) [48]. The catalyst is composed of a prolinamide moiety, responsible for enamine activation of the ketone, and a fumaramide scaffold, which enables hydrogen bonding and, in the interlocked form, positions the macrocycle along the catalytic axis. When tested in the reaction between acetone and *β*‐nitrostyrene, the non‐interlocked catalyst **54** afforded the (*S*)‐enantiomer of the product with an enantiomeric excess of 44%. In contrast, its interlocked analogue **55** reversed the sense of enantioinduction, yielding the (*R*)‐enantiomer in 46% enantiomeric excess. To understand the origin of this enantiodivergence, DFT calculations were performed on simplified enamine intermediates derived from both catalysts. In the case of the non‐interlocked catalyst **54**, DFT calculations showed that both transition states, leading to the (*S*)‐ and (*R*)‐enantiomers (respectively, **TS_54,_ *_Si_*** and **TS_54,_ *_Re_***), exhibit hydrogen bonding between the amide NH group of the catalyst and the nitro group of *β*‐nitrostyrene, with H···O distances of 1.99 Å. The transition state leading to the (*S*)‐product is favored by 7.0 kJ/mol, due to a more favorable spatial orientation between the enamine and the aromatic ring of the nitrostyrene, which allows for reduced steric interactions and an efficient Bürgi–Dunitz‐type approach [49]. In the interlocked (rotaxane) catalyst **55**, hydrogen bonding with the nitrostyrene is observed only in the transition state leading to the (*R*)‐enantiomer (**TS_55,_ *_Re_***). In this case, the hydrogen bond originates from the macrocycle's NH group, with an H···O distance of 1.92 Å and is accompanied by a favorable alignment of the reacting partners. This stabilizes the transition state relative to the alternative (pro‐S) pathway (**TS_55,_ *_Si_***), which lacks such interaction, resulting in a 7.3 kJ/mol preference for the (*R*)‐enantiomer (Scheme 17b).

**SCHEME 18:**
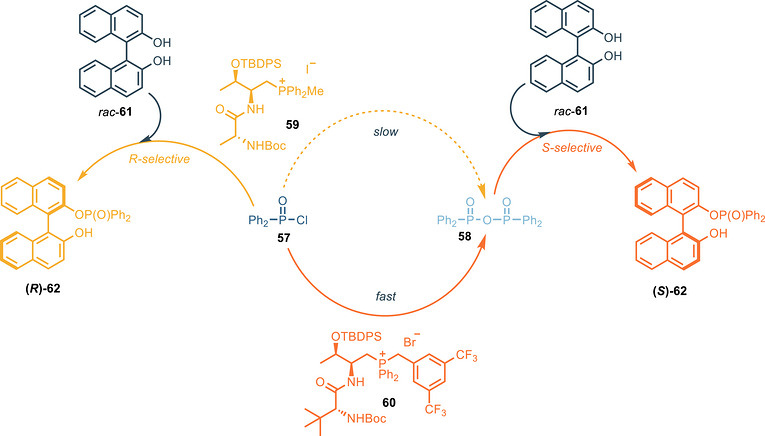
Catalytic process developed by Wang et al., inspired by the AT‐reaction. Two P‐species **57** and **58** are formed as intermediates. Single chiral phosphonium salts can differentiate between **57** and **58**, leading to (*R*)‐ or (*S*)‐selective pathway.

An interesting enantiodivergent process was developed by Wang and coworkers in 2021 for the synthesis of axially chiral biaryl compounds by kinetic resolution (KR) [50]. Dipeptide–phosphonium salts with the same enantiomeric configuration but different P‐substituents and counterion catalyze an Atherton–Todd reaction [51, 52] between phosphine oxides and 1,1′‐biaryl‐2,2′‐diols **61** to provide axially chiral organophosphorus compounds. The highly reactive phosphoryl chloride **57** is generated in situ via base‐promoted halogenation of organophosphine oxides and readily undergoes nucleophilic substitution with alcohols and amines (Scheme 18). In the absence of catalysts, both P‐species **57** and **58** were observed in the reaction mixture by ^31^P‐NMR, whereas their interconversion rates varied in the presence of different phosphonium salts. Phosphonium iodide catalyst **59** slowly promotes the conversion of **57** to **58**, making **57** the catalytically relevant intermediate. In contrast, phosphonium bromide catalyst **60** rapidly converts **57** to **58**, **58** constitutes the active species under catalytic conditions. The authors suggested that enantiodivergence arises from hydrogen‐bonding interactions between the catalyst and the selected P‐species, which constitute the perfect match.

**SCHEME 19:**
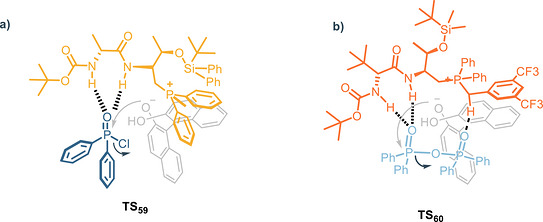
(a) proposed transition state for *R*‐selective cycle and (b) *S*‐selective cycle in the presence of different P‐species intermediate.

To elucidate the origin of catalyst selectivity, a series of control experiments was conducted. When **57** is directly involved in the phosphorylation reaction catalyzed by **59**, the process remains (*R*)‐selective, affording **(*R*)‐62** with high enantioselectivity (99% ee). In contrast, compound 58 under the same conditions leads to the formation of **(*S*)‐62** with a low ee value. An essentially complementary behavior occurs with catalyst **60**: a perfect match takes place when **58** is employed, yielding **(*S*)‐62** with 99% ee, whereas low selectivity is observed in the presence of **57**. As a further validation, the use of optically pure **61** as substrate revealed a clear match‐mismatch relationship: catalyst **59** is well matched with **(*R*)‐61**, rapidly furnishing **(*S*)‐62**, whereas catalyst **60** is perfectly matched with **(*S*)‐61**, yielding **(*R*)‐62** as product. The authors proposed transition state models supported by ^1^H‐NMR titration experiments, rather than theoretical calculations. These studies were carried out on catalysts **59** and **60** in the presence of **57** and **58** as titrating agents, respectively. For catalyst **60**, downfield shift of amide, carbamate, and benzylic protons was observed upon addition of **56**, indicating the formation of three hydrogen‐bonding interactions (Scheme 19a). In contrast, owing to the absence of benzylic protons on catalyst **59**, only amide and carbamate protons participate in hydrogen‐bonding interactions with **57** (Scheme 19b).

**SCHEME 20:**
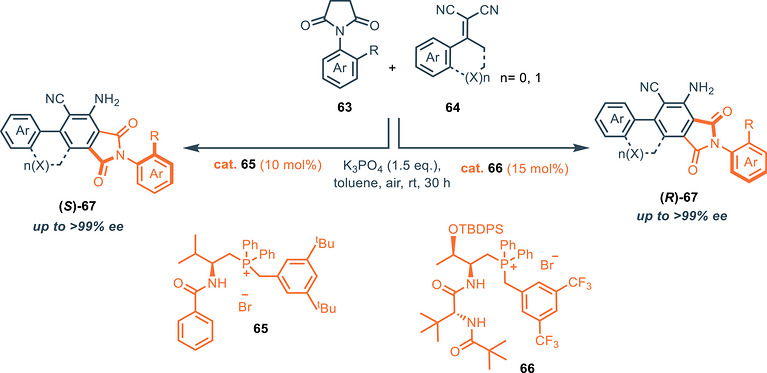
Enantiodivergent synthesis of C─N atropoisomers via [4 + 2] cycloaddition/aromatization catalyzed by phosphonium salt‐based catalysts.

Wang et al. recently reported the application of similar phosphonium salt‐based catalysts for the synthesis of axially chiral *N*‐heterobiaryl compounds exploiting enantiodivergent catalysis with good to excellent enantiocontrol under mild conditions [53]. (*L*)‐Amino acid‐derived bifunctional phosphonium salts were employed for the cascade reaction between *N*‐aryl maleimides **63** and *α*,*α*‐dicyanoolefins **64** using Cs_2_CO_3_ in toluene at room temperature to obtain the desired product **67** as both (*R*) and (*S*) atropoisomers. The two phosphonium salt‐based catalysts **65** and **66** share the same configuration and the same counterion bromide, but they differ in steric hindrance and hydrogen bond donor sites. Specifically, (*L*)‐valine‐derived phosphonium salt **65** catalyzed the formation of **(*S*)‐67** products up to 92% of yield and >99% of ee. On the other hand, (*L*)‐threonine‐derived phosphonium salt **66** catalyzed the formation of **(*R*)‐67** products up to 94% of yield and >99% of ee (Scheme 20). This methodology proves to be highly efficient and with broad functional group tolerance both for the maleimide moiety and *α*,*α*‐dicyanoolefins. The authors conducted mechanistic studies to understand the origin of the enantiodivergent outcome during the cascade. Results conducted at 0°C allow the isolation of a Michael addition intermediate formed through an asymmetric nucleophilic reaction between the two substrates **63** and **64** under basic conditions. The asymmetric induction was transmitted via catalyst‐substrate ion‐pairing and hydrogen bonding formation. ^1^H‐NMR titration in the presence of maleimide revealed different shift changes related to catalysts **65** and **66**, supporting the hypothesis that phosphonium salt‐based catalysts can differentiate between the atropisomeric *Re* and *Si* faces of *N*‐aryl maleimides based on the formation of two different chiral pockets. The authors state that catalyst **65** preferentially forms two hydrogen‐bonding and ion‐pairing interactions with compound **63** (a strong ^1^H‐NMR shift was observed for amide‐NH and benzyl C─H of the catalyst **65** with the titration of substrate **63**). While in the case of catalyst **66**, both substrates **63** and **64** are simultaneously involved through triple‐hydrogen bonding and ion‐pairing interactions (a strong ^1^H‐NMR shift was observed for both amide‐NH and benzyl C─H of the catalyst **66** with the titration of substrate **63**), preferentially approaching the *Si* face of maleimide affording **(*R*)‐67**.

**SCHEME 21:**
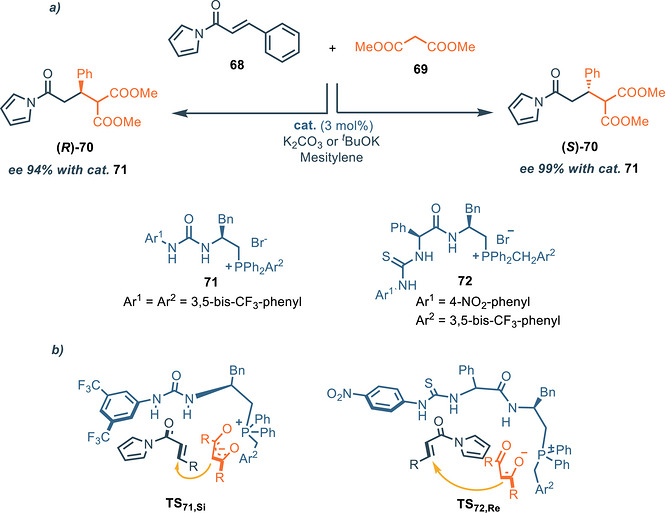
(a) Enantiodivergent Michael addition of malonates to *α*,*β*‐unsaturated amides. (b) Proposed TS for catalyst **71** (*Si*‐attack) and **72** (*Re*‐attack).

Phosphonium salt‐based organocatalysts have also been successfully employed in the enantiodivergent Michael addition of malonates **69** to *α*,*β*‐unsaturated amides **68** [54]. Notably, mono‐ and dipeptidic phosphonium catalysts enable a reversal of enantioselectivity in this transformation. When a phenylalanine‐derived catalyst **71** was used, the corresponding (*R*)‐configured product was obtained in good yield and with satisfactory enantioinduction. In contrast, the use of the dipeptidic phosphonium catalyst **72** led to the formation of the (*S*)‐enantiomer with excellent enantioselectivity (Scheme 21a). This switch in stereochemical outcome is attributed to the distinct hydrogen‐bonding networks established by the two catalyst types. Importantly, the phenylalanine‐based catalyst **71** showed reduced enantioselectivity with aliphatic Michael acceptors, presumably due to steric hindrance affecting the approach of the nucleophile. Conversely, the ditopic dipeptidic catalyst **72** maintained high levels of enantioinduction across a broader substrate scope. Mechanistic investigations and control experiments support a model in which each catalyst organizes the transition state to favor nucleophilic attack on opposite prochiral faces of the electrophile. In the case of the monopeptidic catalyst, the urea moiety engages in hydrogen bonding with the carbonyl of the Michael acceptor, while the phosphonium centre electrostatically interacts with the malonate nucleophile, thereby favoring attack on the *Si*‐face. The presence of bulky aliphatic groups in the Michael acceptor likely disrupts this transition state, leading to diminished selectivity. On the other hand, the dipeptidic catalyst, through a different three‐dimensional arrangement and hydrogen‐bonding network, directs the reactants such that the attack occurs on the *Re*‐face, thus yielding the opposite enantiomer with consistently high enantioselectivity (Scheme 21b).

**SCHEME 22:**
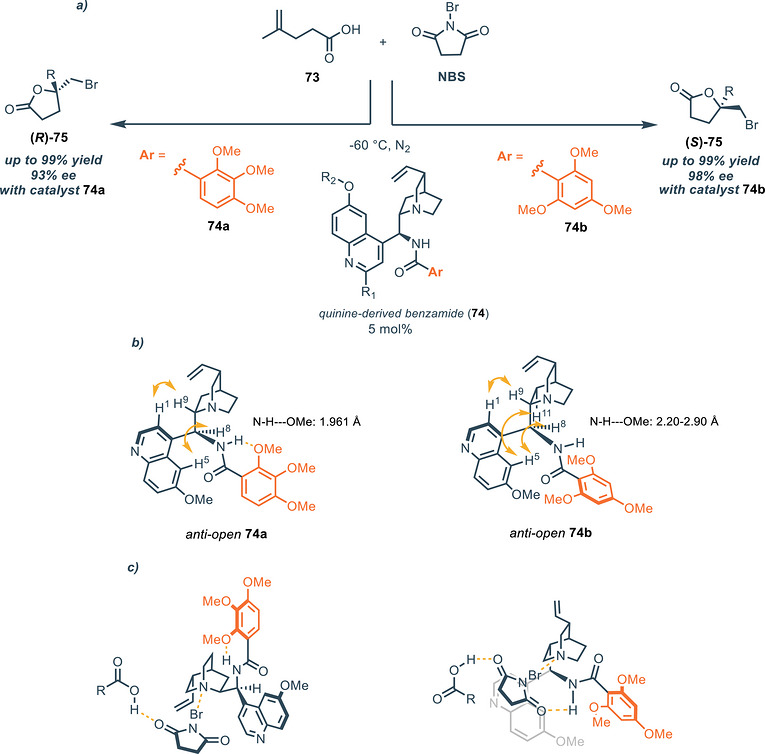
(a) Enantiodivergent bromolactonization of olefinic acids catalyzed by a pair of quinide‐derived benzamide; (b) anti‐open conformation of the two catalysts deduced by NOE studies; (c) different coordination between catalysts and substrates.

Bromolactonization of olefinic acids is another example of enantiodivergence induced by minimal modifications of single chiral catalysts [55]. Yeung and coworkers reported the enantiodivergent bromolactonization of compound **73** using *N*‐bromosuccinimide (NBS) as the brominating source. The reaction is catalyzed by quinine‐derived catalysts, differing in the substitution pattern on the aromatic moiety, at −60°C, under inert atmosphere and in the absence of light (Scheme 22a). Enantioselectivity is switched when the methoxy substituent is on a different position on the aryl amide of quinine‐derived benzamide. Both catalysts afford the desired product in good yields and high enantiomeric excess. Structural analysis and DFT calculations were carried out on **74a** and **74b** to understand the origin of different behavior in catalysis. Based on the NOE correlations, both catalysts are in an anti‐open conformation (Scheme 22b). Nevertheless, a slight difference exists between the two geometries. In the case of **74a**, an intramolecular hydrogen bond is formed between N─H and *ortho*‐OMe of the 2,3,4‐trimethoxyphenyl moiety. While in the case of **74b**, the intramolecular interaction between N─H and *ortho*‐OMe of the 2,4,6‐trimethoxyphenyl moiety is weak because of steric hindrance. Also, in this case, noncovalent interactions seem to be the key for enantiodivergence, because the intramolecular hydrogen bond leads to different coordination towards substrate **73** and NBS. The free energy profile shows that for catalyst **74a**, the amide's N─H is not involved in the catalytic process because the intramolecular hydrogen bond restrains the aryl amide moiety. Thus, the 2,3,4‐trimethoxyl benzamide acts mainly as a steric shielding group. On the other hand, for catalyst **74b**, the amide's N─H can actively coordinate NBS, and the enantioselectivity is controlled by both amide and quinuclidine groups (Scheme 22c). The crucial role of hydrogen bonding interactions was also demonstrated by *N*‐methylated benzamides analogues, which furnished the desired product **75** with dramatically low ee in both cases. Interestingly, chiral reverse induction is lost because the product is obtained with the same configuration **(*R*)‐75** in both cases [55].

**SCHEME 23:**
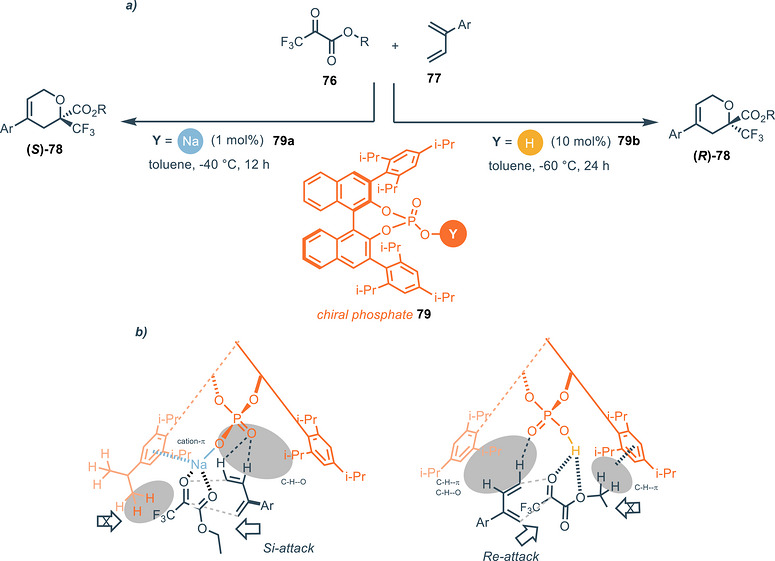
(a) Enantiodivergent oxa‐Diels–Alder between trifluoropyruvate and simple dienes; (b) proposed stereoselective models, which explain the origin of the enantiodivergent process. Adapted from ref [56].

In the same context, Zhang, Luo, and coworkers have recently reported an enantiodivergent oxa‐Diels‐Alder reaction catalyzed by a single chiral phosphoric acid (CPA) [56]. Trifluoropyruvates **76** smoothly react with simple dienes **77** to obtain chiral dihydropyrans **78** as both (*R*) and (*S*) isomers by switching from the protonated form **79b** and deprotonated one **79a** with Na^+^ as counter ion (Scheme 23a). The authors were interested in chiral sodium phosphate catalyst **79a**, which provided good results both in terms of yield and selectivity among different chiral phosphate salts. By carrying out control experiments with free phosphoric acid analogue **79b**, the chiral induction was completely reversed even if the catalyst configuration was unchanged. DFT calculations were conducted to understand the origin of enantiodivergent catalysis, and concerted over stepwise processes were considered for the oxa‐Diels‐Alder reaction with ethyltrifluoropyruvate. Stereoselectivity seems to be determined mainly by weak noncovalent interactions like π‐interactions and dispersion forces (Scheme 23b). Specifically, in **79a**, chirality inversion is induced by sodium cation‐ π interaction with the aryl ring of **79a**, which determines a *C*2‐symmetry distortion, creating an unsymmetric chiral pocket that assembles the substrates through the Si‐attack of diene on trifluoropyruvate. On the other hand, **79b** requires more demanding reaction conditions to ensure compound **78** in good yield and enantioselectivity: lower temperature (−60 °C instead of −40 °C), longer reaction time (24 h instead of 12 h) and higher catalyst loading (10 mol% instead of 1 mol%) lead to **(*R*)‐78** up to 99% yield and ee. With **79b** two bidentate hydrogen‐bonding with both trifluoropyruvate and diene were established, a stronger one O─H which coordinates trifluopyruvate, and a weak C─H interaction with diene, leading to favored *Re*‐facial selectivity.

## 5 Other Strategies to Achieve Enantiodivergence

**SCHEME 24:**
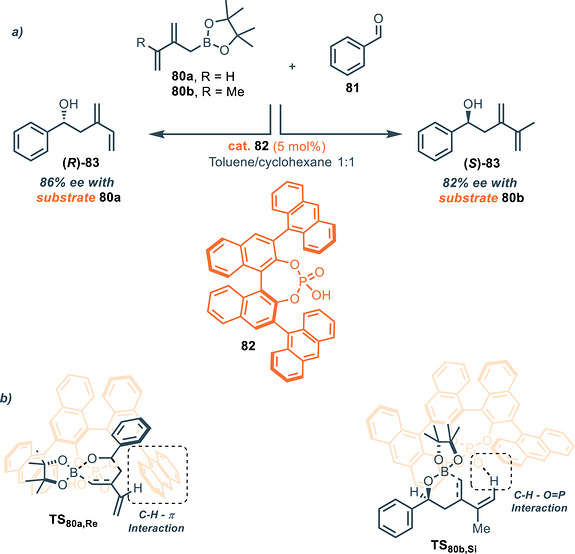
(a) Enantiodivergent allylation of benzaldehyde with β‐alkenyl allylic boronates. (b) Transition state for the two substrates **80a** and **80b**.

It is widely demonstrated that minimal modifications to the catalyst can lead to dramatic differences in the enantiomeric outcome of a reaction [1, 3]. However, enantioselectivity reversal caused by small structural changes in the substrate structure has been comparatively underexplored. In this context, in 2022, Chen and coworkers reported the chiral phosphoric acid (CPA)‐catalyzed asymmetric allylation of β‐alkenyl allylic boronates **80** with aldehydes **81** to afford enantioenriched homoallylic alcohols **83** (Scheme 24a) [57]. Interestingly, the authors observed that subtle changes in the substrate structure had a profound impact on the sense of asymmetric induction. When the allylic boronate bears a vinyl group (i.e., *β*‐vinyl‐substituted allylic boronate, R = H, compound **80a**), the reaction proceeds with high enantioselectivity, yielding the (*R*)‐configured alcohol **(*R*)‐83**. In contrast, when the *β*‐position is substituted with a 2‐propenyl group (β‐2‐propenyl‐substituted allylic boronate, R = Me, compound **80b**), the opposite enantiomer, (*S*)‐configured alcohol **(*S*)‐83**, is obtained, despite using the same chiral catalyst. To rationalize this unusual enantiodivergence, the authors performed DFT calculations to analyse the favored transition states for each substrate. In the case of **80a**, the preferred transition state (**TS_80a,Re_**) involves a re‐face attack on the aldehyde, stabilized by a C─H···π interaction between the vinyl group of the substrate and the 9‐anthracenyl substituent of the catalyst. However, when using **80b**, the additional methyl group disrupts the geometry required for this stabilizing interaction. As a result, C─H···π interactions are no longer significant. Instead, the methyl group introduces steric repulsion in the catalyst pocket, destabilizing the transition state that would lead to the (*R*)‐product. The favored transition state for this substrate (TS‐**_80b,Si_**) thus involves a *Si*‐face attack on the aldehyde, benefiting instead from a reinforcing C─H···O = P hydrogen bond with the phosphoryl oxygen of the catalyst (Scheme **24b**). This subtle change in noncovalent interactions accounts for the observed inversion of stereochemical outcome.

**SCHEME 25:**
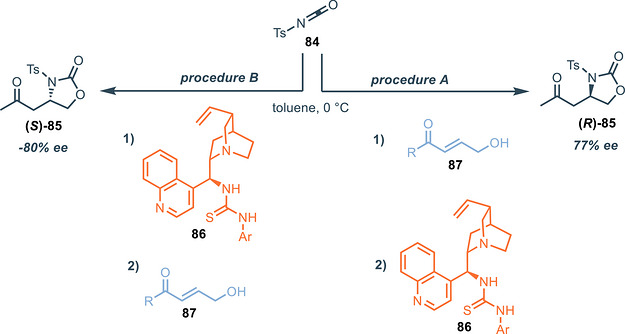
Enantiodivergent procedure for the organocatalytic synthesis of 2‐oxazolidinones by switching the addition order of substrates and catalyst.

In some cases, the order of addition can induce enantiodivergence without any change in the reaction components. This phenomenon is particularly intriguing because no synthetic modification of either the reagents or the catalyst is required. Due to the scarcity of examples describing enantiodivergence arising from the order of reagent addition, rather than from structural modifications of the catalyst or substrates or from more conventional variations in reaction conditions, an earlier study is included here as a representative case. Asano, Matsubara et al. reported in 2013 an unexpected enantiodivergent [3+2] cyclization catalyzed by chinchona derivatives [58]. Enantiodivergence mainly depends on the catalyst addition. **(*R*)‐85** is obtained in modest yield when isocyanate **84** reacts first with γ‐hydroxy‐α,β‐unsaturated carbonyl compound **87** and then catalyst **86** is added (Scheme 25, procedure A). On the other hand, when **86** and isocyanate **83** are mixed first, and compound **86** is added after, the desired product is obtained as **(*S*)‐85** in good yield (Scheme 25, procedure B). On the model substrate, procedure A yields 77% ee, while procedure B −80% ee. Although the enantioselectivity achieved is not very high, Asano and Matsubara's work represents one of the first examples of reversal enantioselectivity without any change in reaction components or structural catalyst changes, but only timing in the components addition. However, the mechanism governing the observed enantiodivergence has not been clarified. The authors only identified the formation of a 1:1 complex between the organocatalyst and the isocyanate, whose presence is presumed to favor the formation of the (S)‐enantiomer, whereas catalyst addition *after* the reaction components leads predominantly to the formation of the *(R)*‐enantiomer.

## 6 Summary and Outlook

The analysis of the literature published from 2018 to date illustrates that enantiodivergent organocatalysis has evolved from isolated empirical observations into a versatile synthetic strategy, enabling access to both enantiomers through solvent‐mediated entropic/enthalpic modulation, the engagement of noncovalent interactions via achiral additives, or minimal steric and electronic perturbations of the catalyst structure. Nevertheless, despite these methodological advances, the field faces critical bottlenecks that hinder its systematic application, primarily the continued dominance of serendipity over rational design; the subtle free energy differences required to invert stereoselectivity are often difficult to engineer a priori without extensive trial‐and‐error screening. A major challenge lies in the limitations of static DFT calculations, which frequently struggle to fully capture the complexity of systems driven by entropy, solvation, and dispersion forces, necessitating an urgent shift toward the systematic integration of Molecular Dynamics (MD) simulations and explicit solvation models to understand pre‐reactive complex dynamics before synthesis is undertaken. Furthermore, the lack of general robustness, where minor substrate variations can nullify or reverse selectivity, remains a significant drawback for broader applications, highlighting the need for more robust catalytic systems. To overcome these limitations, future research must embrace multidisciplinary approaches, potentially utilizing Chemoinformatics and Machine Learning to model the nonlinear interactions between solvents and catalysts, while simultaneously exploring novel activation modes such as mechanically interlocked molecules or dynamic supramolecular assemblies to finally make enantiodivergence a rationally designed feature of asymmetric synthesis.

## Conflicts of Interest

The authors have no conflicts of interest.

## Data Availability

Data sharing not applicable to this article as no datasets were generated or analyzed during the current study.
